# Novel Approaches in Mycotoxins Research: Detection, Prevention and Mode of Action

**DOI:** 10.3390/toxins17040161

**Published:** 2025-03-24

**Authors:** Ana Juan-García, Daniela Jakšić

**Affiliations:** 1 Department of Preventive Medicine and Public Health, Food Sciences, Forensic Medicine and Toxicology, University of Valencia, 46100 Burjassot, Valencia, Spain; 2 Department of Microbiology, Faculty of Pharmacy and Biochemistry, University of Zagreb, 10000 Zagreb, Croatia; djaksic@pharma.unizg.hr

Mycotoxins, which are toxic secondary metabolites produced by fungi, pose significant risks to food safety and public health. The research included in this Special Issue primarily focused on the regulated mycotoxin aflatoxin B1 (AFB1), fumonisins, and ochratoxin A (OTA), addressing their toxic effects, detection methods, and potential mitigation strategies. The highlights of this research include the genetic basis of fumonisin resistance in *Fusarium verticillioides* (Krska et al.), the development of a validated HPLC-FLD method for the quantification of ochratoxin A in models of mice neurotoxicity (Beraza et al.), and the assessment of mycotoxin contamination in hops when used in brewing (Sarkanj). Advances in machine learning for the rapid detection of mycotoxins (Inglis et al.) and protective compounds such as quercetin (Pauletto et al.) and functional food ingredients, e.g., fermented whey (Trombetti et al.), provide novel insights into the mitigation of toxins. Studies on polyphenolic plant extracts (Cadenillas et al.) also highlight eco-friendly antifungal alternatives.

As described in Krska et al. (2024), *Fusarium verticillioides* produces fumonisins, which inhibit the biosynthesis of sphingolipids in various organisms. These mycotoxins function as virulence factors in plant pathogens and influence interactions between competing fungi. It is reported that fumonisin-producing *Fusarium verticillioides* exhibits higher resistance to fumonisin B1 (FB1) compared to non-producing *F. graminearum*. By investigating the genetic basis of this resistance, Krska and colleagues found that the overexpression of certain ceramide synthases, particularly FUM18, conferred a high level of resistance, suggesting that *F. verticillioides* possesses a redundant self-resistance mechanism.

Similarly, Beraza et al. (2024) underscore the dangers of ochratoxin A (OTA), a mycotoxin that is commonly found in food products and has been increasingly linked to neurodegeneration. However, despite its relevance, no fully validated HPLC analytical methods are currently available for the quantification of OTA in mice, which is used as an animal model in neurotoxicity research. In order to address this gap, Beraza’s team developed a highly sensitive and robust HPLC-FLD method that was validated according to FDA and EMA guidelines. This methodology enables the precise quantification of OTA in crucial tissues such as the brain and intestine, enabling more accurate neurotoxicity studies.

Meanwhile, Sarkanj provides insights into mycotoxin contamination in hops, a crucial ingredient in brewing. Analyzing 62 hop samples from Croatian craft breweries, the study confirmed that the hops had been contaminated with *Alternaria* and *Fusarium* toxins. Tenuazonic acid was detected in all samples, while deoxynivalenol appeared in 98% of them. However, the absence of *Aspergillus* and *Penicillium* toxins indicated that appropriate storage conditions were being employed. Additionally, regional differences in toxin levels highlighted the necessity for targeted monitoring to ensure the safety of hops in the brewing and pharmaceutical industries.

In another study, Inglis et al. (2024) explore the application of machine learning (ML) in mycotoxin detection. Traditional laboratory analyses, while effective, are time-consuming and impractical for large-scale screenings. ML applications have emerged as promising alternatives, offering accuracy and efficiency. Inglis reviews recent ML models used for mycotoxin detection, noting that neural networks, particularly convolutional ones, dominate the field. However, challenges remain, including the lack of detailed reporting on hyperparameters and open-source code, which hampers reproducibility and the optimization of models.

Expanding on mycotoxin-induced toxicity, Pauletto et al. (2023) investigate the protective role of quercetin (QUE) against aflatoxin B1 (AFB1) in bovine fetal hepatocyte-derived cells. AFB1 induces oxidative stress, inflammation, and transcriptional changes linked to carcinogenesis. Encouragingly, QUE reduced AFB1-induced cytotoxicity and the peroxidation of lipids, exerting broader transcriptional modifications than AFB1 alone. Notably, QUE reversed AFB1-induced alterations in the enzymatic activity of CYP3A, further supporting its protective role. These findings pave the way for in vivo studies that explore the potential role of QUE in mitigating aflatoxicosis.

Additionally, Trombetti et al. (2025) examine the hepatotoxic effects of exposure to AFB1 and OTA in Wistar rats and evaluate the mitigating properties of fermented whey (FW) and pumpkin (P). Proteomic analysis revealed the significant downregulation of differentially expressed proteins (DEPs) in the presence of AFB1 and OTA, suggesting a synergistic toxic effect. However, FW and P supplementation helped counteract these harmful effects, underscoring their potential application as functional ingredients in mitigating mycotoxin-induced damage.

Exploring alternative protective strategies, Cadenillas et al. (2024) investigate the antifungal effects of plant extracts against AFB1. Extracts from *Annona muricata* and *Uncaria tomentosa* inhibited the synthesis of AFB1 in a dose-dependent manner, correlating with their polyphenol content. More specifically, catechin and epicatechin played crucial roles in the inhibition of AFB1, with catechin reducing the production of toxins by 45% at concentrations comparable to the extracts. These findings highlight the potential use of plant-derived compounds as eco-friendly alternatives to synthetic fungicides.

Cherewyk et al. (2023) and Sá et al. (2024) address concerns regarding the stability and absorption of mycotoxins in food products. Cherewyk’s research on ergot alkaloids in wheat reveals that the total ergot concentration fluctuates over time, emphasizing the need for proper storage and timely quantification. Meanwhile, Faria examines the bioaccessibility of AFB1, enniatin B, and sterigmatocystin in breakfast cereals. Notably, milk significantly influenced the bioaccessibility of mycotoxins, with variations depending on the milk type. These insights stress the importance of studying co-occurrence and dietary interactions to assess the exposure of humans to mycotoxins comprehensively.

Lastly, in a review article, Bridgeman et al. (2024) examine the neurological effects of acrylamide (AA) and mycotoxins, which frequently co-occur in food matrices such as cereals and coffee. With a focus on the SH-SY5Y neuroblastoma cell model, this study analyzed the cytotoxicity, apoptosis, oxidative stress, and axonopathy induced by AA and mycotoxins over the past decade. Bridgeman highlighted the growing scientific interest in studying their combined effects, as well as the need for further research into mitigation strategies. Notably, while bioactive compounds in food have been shown to counteract the toxicity of mycotoxins, there remains a significant gap in the knowledge regarding AA, underscoring the need for continued investigation.

While these findings provide crucial insights into the detection of mycotoxins, their resistance mechanisms, and mitigation strategies, several questions remain. Standardized ML models and the long-term effects of food contaminants combined with mycotoxins require further exploration. Additionally, the stability of mycotoxins in stored food and the potential for bioactive compounds to counteract toxicity warrant deeper investigation.

The most critical gap in mycotoxin research is the lack of comprehensive data regarding human exposure and validated biomarkers for risk assessment. While analytical advancements, such as the validated HPLC-FLD method for OTA in mice (Beraza et al.), provide a foundation, there remains a significant need for standardized, sensitive detection methods that can be employed in human biological matrices (e.g., blood, urine, tissues). Without robust biomonitoring tools, the assessment of real-world exposure levels and long-term health effects remains challenging. Additionally, the interplay between mycotoxins and food components, as well as their cumulative and synergistic toxic effects (e.g., with acrylamide, as highlighted by Bridgeman et al.), are underexplored. The limited use of advanced human-relevant models, such as organoids and co-culture systems, also hinders a deeper mechanistic understanding of mycotoxin toxicity in humans. Addressing these gaps through interdisciplinary research that combines analytical chemistry, toxicology, and computational modeling will be crucial for improving risk assessments and developing targeted mitigation strategies.

